# Alport Syndrome is a Partial Tubulointerstitial Disease of the Kidney

**DOI:** 10.1016/j.ekir.2025.11.019

**Published:** 2025-11-17

**Authors:** Lisa Loderbauer, Karl X. Knaup, Daniel Reisenbüchler, Nicolas Kaiser, Stephanie Naas, Karen Schneider, Florian J. Wopperer, Antje Wiesener, Francesca Pasutto, Mario Schiffer, Christoph Daniel, Katharina A.E. Broeker, Dorit Merhof, Maike Buettner-Herold, Michael S. Wiesener

**Affiliations:** 1 Department of Nephrology and Hypertension, University Hospital Erlangen, Erlangen, Germany; 2 Department of Image Analysis and Computer Vision, University of Regensburg, Regensburg, Germany; 3 Research Center On Rare Kidney Diseases, Else Kröner-Fresenius-Stiftung and the Eva Luise und Horst Köhler Stiftung, Erlangen, Germany; 4 Department of Nephrology, Center for Rare and Genetic Kidney Diseases, TUM University Hospital Rechts der Isar, TUM School of Medicine and Health, Munich, Germany; 5 Institute of Human Genetics, University Hospital Erlangen, Erlangen, Germany; 6 Department of Nephropathology, University Hospital Erlangen, Erlangen, Germany; 7 Institute of Physiology, University of Regensburg, Regensburg, Germany

**Keywords:** extracellular matrix, hearing impairment, hereditary nephritis, microhematuria, proteinuria, thin basement

## Abstract

**Introduction:**

Recent genetic studies have shown that Alport syndrome (AS) is much more prevalent than clinically recognized, suggesting that atypical cases may phenocopy other kidney diseases. To date, pathomechanistic studies of AS have focused exclusively on the glomerular membrane, yet equally strong expression of collagen α(IV) chains is found along the distal renal tubule. We hypothesized that genetically determined abnormality of the tubular collagen IV (α345) molecule contributes to kidney failure and may drive atypical phenotypes.

**Methods:**

Histology and primary tubular cells (PTCs) of 8 patients with AS were investigated alongside controls.

**Results:**

Collagen α5 (IV) was detected within the tubular basement membrane (BM) (TBM) of the distal segments of renal tubules by immunohistochemistry. *In situ* hybridization on human tissues and protein detection of collagen α5 (IV) in PTC cultures clearly showed that the distal tubular apparatus predominantly produces collagen IV for the TBM. Electron microscopy of biopsies from patients with AS demonstrated irregularities of the TBM, somewhat similar as described for the glomerular BM (GBM). Finally, computer-assisted analyses showed that in biopsies of patients with AS, interstitial fibrosis preferentially occurs in spatial vicinity of the affected distal tubules.

**Conclusion:**

Our study demonstrates that the collagen IV (α345) molecule within the TBM is largely produced by the distal tubule itself. In AS, the TBM shows ultrastructural changes, which may induce fibrotic molecular signatures, as tubulointerstitial fibrosis appears to start in the vicinity of the distal tubule. Therefore, we postulate that the progression of kidney disease in AS may in part stem from the (distal) tubular apparatus.

## Graphical abstract

**Figure:**
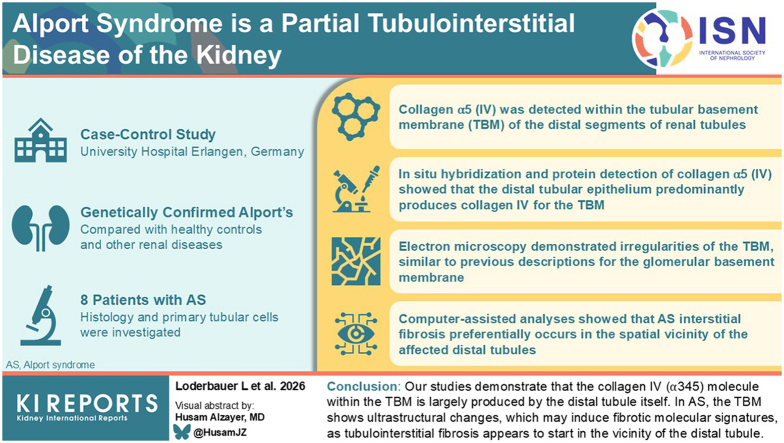

BMs are sheet-like structures of the extracellular matrix which provide biomechanical support and a signaling interface of cells with their environment. The BM is a multiprotein network of components such as laminin, perlecan, and nidogen, where cellular adhesion is partially achieved by interaction with integrins and α-dystroglycans.^1^^,^^2^ A major component of any BM is collagen type IV, which consists of heterotrimers of distinct IV (α) chains secreted as protomers and then assembled to a supramolecular structure in the extracellular space.^3^

In the kidney, the collagen IV molecular network is a functionally important component of the GBM, which spatially separates podocytes from endothelial cells, while itself critically contributing to the glomerular filtration barrier.^4^ Disease-causing germline variants in any of the *COL4A3*, *COL4A4*, or *COL4A5* genes have the potential to disrupt the integrity of the GBM, often causing microscopic hematuria and proteinuria in early life and then progressing to chronic kidney disease.^5^^,^^6^

Already, the very first report of immunodetection of the collagen IV (α345) molecule in the kidney, using serum of a patient with AS and anti-GBM disease in his kidney transplant, described staining of the TBM, next to the GBM.^7^ Knowledge of the respective genes enabled generation of specific antibodies against their products and thereby careful characterization in numerous reports of renal expression in the 1990s.^8^, ^9^, ^10^ In addition, these studies demonstrated developmental changes in terms of an isotype switch from fetal to adult composition of the BM (predominantly α1α1α2 to α3α4α5 heterotrimer), which in the TBM appear to be very similar to the GBM, although the latter has been studied much more carefully. The same appears to be true for the formation of the TBM in AS, where the collagen IV (α345) molecule is absent or diminished and the collagen IV (α112) molecule predominates. In the GBM, this leads to decreased mechanical stability of the membrane,^11^ which is presumed to be an early mechanism of glomerular pathology. To date, no data exist on the putative consequences of these changes in the TBM of AS.

Systematic exome sequencing of large patient cohorts with chronic kidney disease has shown that AS is much more frequent than previously thought.^12^ The reasons for not correctly diagnosing patients with genetic diseases can be numerous, but may be partially caused by phenotypic variability. AS can phenocopy other glomerular diseases such as podocytopathic focal segmental glomerulosclerosis^13^ as reviewed in Deltas *et al.*,^14^ or even be clinically misclassified as tubulointerstitial disease.^15^^,^^16^ We hypothesized that disruption of the collagen IV molecule in the TBM can influence the clinical phenotype and possibly contribute to its variability, perhaps to disease progression. Therefore, improved pathological or physiological knowledge of the collagen IV molecule in the tubulointerstitial space may improve our understanding of AS, and influence pharmacokinetic design of putative targeted drug development.

Our study characterizes the collagen IV (α345) molecule in human kidney, describing its exact localization and identifying the distal tubular, epithelial cell as being predominant in collagen synthesis. Moreover, analysis of kidney biopsies from patients with AS demonstrates irregularities of the distal TBM, as well as spatial induction of fibrosis which implicates a role in the pathomechanism of the disease.

## Methods

### Patient and Ethical Approval

All patients provided written informed consent to clinical and scientific procedures. The studies were approved by the ethics committee of the Friedrich-Alexander University Erlangen-Nürnberg (approval number: 251_18 B). Criteria for monocentric patient selection with AS was on the basis of existing routine genetic reports and the existence of a kidney biopsy. All kidney biopsies investigated were performed between 2007 and 2021 for purely clinical criteria and not for the present study.

### Cell Culture

If not stated otherwise, all reagents were purchased from Sigma-Aldrich (Taufkirchen, Germany). HeLa cells were supplied from the German Collection of Microorganisms and Cell Cultures (DSMZ, Braunschweig, Germany). The following reagents were used for culture: DMEM, 1.0 g glucose/l, 10% fetal calf serum, 2 mmol/l L-glutamine, 100 U penicillin, and 100 μg streptomycin/ml. Medium as well as penicillin or streptomycin were obtained from PAN-Biotech (Aidenbach, Germany) and fetal calf serum (standard “Gold”) from PAA Laboratories (Coelbe, Germany).

Human PTCs (huPTC) were generated and cultivated as described by Zhou *et al.*^17^ Cells were used for protein or RNA extraction after approximately 3 to 4 passages. The siRNA knockdown was performed on huPTC and HeLa cells over 72 hours according to the manual described by Warnecke *et al.*^18^ The following siRNAs (GeneGlobe IDs given in parentheses) were used: siCOL4A5_5 (SI03124212), siCOL4A5_6 (SI04141935), siCOL4A5_7 (SI04240243), and siCOL4A5_8 (SI04342016) (FlexiTube GeneSolution GS1287, Qiagen, Hilden, Germany).

Dipyridyl was added in a concentration of 100 μM for 18 hours on huPTC and HeLa cells. Detailed methods for immunohistochemistry, colocalization studies and analysis of BMs by electron microscopy are provided in the Supplementary Methods. Information on primary and secondary antibodies can be found in the Supplementary Table S1.

### Immunoblotting

For whole cell extracts, cells were lysed by sonication into extraction buffer (8 M urea, 10% glycerol, 1% SDS, 10 mM TrisHCl pH 6.8, protease inhibitor cOmplete [Roche, Mannheim, Germany]). Protein concentrations were measured with the DC Protein Assay (BioRad, CA) according to the manufacturer’s instructions. Protein separation was performed by SDS-PAGE and proteins were transferred to a polyvinylidene fluoride membrane (Millipore, Bedford, MA). Five percent milk in TBS-T was used as blocking buffer for 1 hour at room temperature after the transfer. For protein detection, a primary antibody against collagen α5 (IV) was incubated at 4 °C overnight, followed by an HRP-conjugated secondary antibody at room temperature for 30 minutes. Details of the antibodies are shown in Supplementary Table S1. Between incubations, the membranes were washed 3 × 5 minutes with TBS-T and after incubation with the secondary antibody 4 × 10 minutes with TBS-T and 2 minutes with phosphate-buffered saline. Signals were visualized using an ECL system (GE Healthcare, Munich, Germany).

### Polymerase Chain Reaction

Cells were homogenized into TRK lysis buffer (peqGOLD Total RNA Kit, VWR life science, Darmstadt, Germany) for RNA extraction.

For reverse transcription, cDNA synthesis was performed with high capacity cDNA reverse transcription kit from Thermo Fisher Scientific, according to the manufacturerʼs instructions with 100 ng RNA.

The reverse transcription was performed in a thermal cycler using the following incubation conditions: 25 °C for 10 minutes, 37 °C for 2 hour, 85 °C for 5 minutes.

For quantitative polymerase chain reaction (PCR) the Maxima SYBR Green/ROX quantitative PCR Master Mix (Thermo Fisher Scientific) was used according to the manual with 2 μl cDNA. After 5 minutes of centrifugation at 4 °C and 1000 rpm, the PRC was performed in StepOnePlus Real-Time PCR System (Applied Biosystems, Waltham, MA).

Incubation steps for PCR were as follows: denaturation at 95 °C for 15 minutes; 30 ×: denaturation at 95 °C for 1 minute; annealing at 58 °C for 1 minute; extension at 72 °C for 45 seconds; final extension: at 72 °C for 10 minutes.

Primers used (at 10 nM) for COL4A5 were as follows:

F: 5‘-CCA GGA ATA CCA GGT CCT AAA G-3‘

R: 5‘-GGA AGA CCT ACA TCA CCA TCT C-3‘

### *In situ* Hybridization

Five μm sections of formalin-fixed paraffin-embedded human kidney biopsies were analyzed using RNAscope Multiplex Fluorescent V2 Assay with RNAscope probes Hs-COL4A5 (Cat. No. 461871) and Hs-CALB1-C2 (Cat. No. 422161-C2) as described in Wang *et al.*^19^ and Broeker *et al.*^20^

### (Re-)Analysis of Single-Nucleus RNA-Sequencing Data

Single-nucleus RNA–sequencing data of control kidneys published by Hinze *et al.*^21^ were downloaded as cellranger-mapped count data and metadata, including cell-type assignments from GEO (GSE210622). Data were processed as previously described using the R package Seurat (version 4.1.0).^22^ For each cell type and individual, a pseudobulk object was computed on raw counts and subsequently normalized to counts per million. Data points show the normalized pseudobulk expression of the indicated RNA transcript of interest per cell type and individual.

### Computer-Assisted Automated Fibrosis Mapping

Formalin-fixed paraffin-embedded kidney biopsies from patients diagnosed with AS (Table 1) were sectioned consecutively at 1.0-μm thickness and stained with either E-cadherin or Masson’s Trichrome. Distal tubules were identified through E-cadherin positivity, whereas proximal tubules were classified based on E-cadherin negativity. In whole slide images stained with Masson’s Trichrome, all tubules were manually annotated and labelled as distal or proximal according to their corresponding E-cadherin staining. For each annotated tubule, a 512 × 512–pixel tile was extracted around the center of each tubule to define a search area for detecting surrounding fibrosis. A green color threshold was manually selected by visual inspection, ensuring consistent fibrosis identification by excluding background and nonfibrotic regions (Figure 6a). Fibrosis was quantified as a metric based on the percentage of image area showing fibrosis after color filtering. To test the hypothesis that fibrosis is more prominent around distal tubules than proximal tubules, statistical analysis was performed with an independent *t* test and Mann-Whitney U test using the outcomes of the fibrotic metric across images.

**Table 1:** Probands with Alport syndrome

Proband ID	Sex	Collagen IV variant	Biopsy	CKD	Age	IF/TA	IHC	EM	AFM	Primary Cells	
										Immunoblot	qPCR
AS001	f	*COL4A4*, Intron 45: c.4333+2T>C, het. (ACMG 4)	+	G4A3	46	30%–40%	+	+	+	-	-
AS002	m	*COL4A5*, Exon 49: c.4418A>G, p.(His1473Arg), hem. (ACMG 4)	+	G3bA3	44	50%–60%	+	+	+	-	-
AS003	m	*COL4A5*, Exon 50: c.4630T>C, p.(Trp1544Arg), hem. (ACMG 4)	+	G3aA3	23	25%–30%	+	+	-	-	-
AS004	m	*COL4A5*, Exon 51: c.4729_4730del, p.(His1577Glufs∗62), hem. (ACMG 4)	+	G1A3	4	< 5%	+	+	-	-	-
AS005	m	*COL4A5*, Exon 25: c.1871G>A, p.(Gly624Asp), hem. (ACMG 5)	+	G3aA3	38	< 5%	+	+	+	+	+
AS006	m	*COL4A5*, c.(276+1_277-1)_(438+1_439-1)dup (Exons 5,6,7), hem. (ACMG 4)	-	G1A3	-	n.a.	-	-	-	+	+
AS007	f	*COL4A5*, c.(276+1_277-1)_(438+1_439-1)dup (Exons 5,6,7), het. (ACMG 4)	-	G1A2	-	n.a.	-	-	-	+	+
AS008	f	*COL4A5*, Exon 29: c.2252del, p.(Pro751Glnfs∗41), het. (ACMG 4)	+	G3aA3	35	15%–20%	+	-	+	-	-

AFM, automated fibrosis mapping; CKD, chronic kidney disease; EM, electron microscopy; f, female; IF/TA, interstitial fibrosis and tubular atrophy; IHC, immunohistochemistry; m, male; qPCR, quantitative polymerase chain reaction.

Proband’s pseudonym (ID) and genetic findings, as well as their clinical data in respect to the date of biopsy (stage of CKD, age and grade of IF/TA) are listed. On the righthand side, the applications are shown, for which purpose each proband's material was analyzed (IHC, EM, AFM, and primary cells for immunoblotting or qPCR).

Image annotations were performed using QuPath (v0.5.1), with labelled tubules exported as GeoJSON files for downstream analysis. All image processing was conducted in Python (v3.11.4) using SlideIO (v2.2.0), with NumPy (v1.25.2) and OpenCV-python (v4.7.0) used for fibrosis quantification and SciPy (v1.11.1) for statistical analysis. Plots were generated with Matplotlib (v3.7.2).

## Results

We investigated kidney biopsies and PTCs of 8 patients with genetically confirmed AS (Table 1), next to healthy controls and other renal diseases.

### Localization of the Collagen α (IV) Chains in Human Kidney

**Figure 1:**
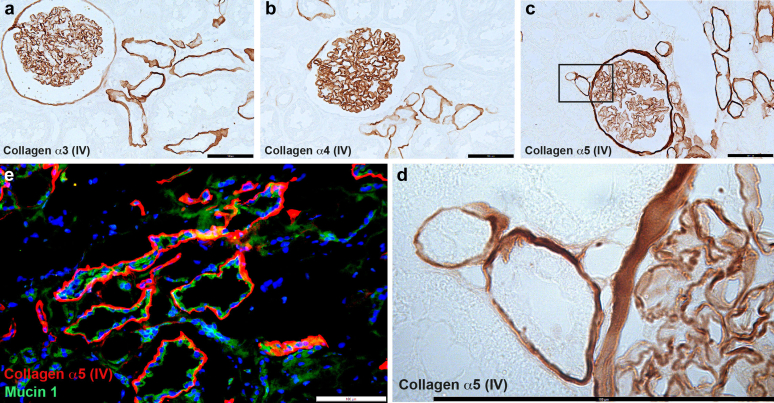
Collagen IV (α345) subunits are located in the basement membrane next to distinct tubular segments Immunohistochemistry for (a) collagen IV α3, (b) α4, and (c) α5 on healthy human kidney tissue sections showing distinct linear immunoreaction of the GBM and the TBM of defined segments. (d) The insert in (c) is enlarged to show representatively that the α5 (IV) protein is exclusively extracellular clearly visible in the TBM, the GBM and the Bowman capsule. (e) Immunofluorescence for collagen α5 (IV, red) and mucin 1 (green), a well-recognized marker of distal tubular segments of the kidney, shows that there is very good, although not complete, overlap of stained tubular segments. Because mucin 1 is situated predominantly apical and collagen α5 (IV) extracellular in the TBM, there is almost no colocalization on a subcellular level of the epithelium (yellow). Scale bars each correspond to 100 μm. GBM, glomerular basement membrane; TBM, tubular basement membrane.

To validate the published data of the 1990s, we stained human kidney tissue for all 3 components of the collagen IV (α345) molecule (Figure 1). As reported previously, the collagen IV a3a4a5 chains are clearly visible in the GBM, as well as the TBM of distinct tubular segments (Figure 1a–d). Interestingly, the Bowman capsule stains positive only for collagen α3 (IV) (Figure 1a) and collagen α5 (IV) (Figure 1c), but not collagen α4 (IV) (Figure 1b). At higher magnification, TBM staining shows a clear linear pattern, with tubular cells completely unstained (Figure 1d). Double-labeling immunofluorescence with collagen α5 (IV) and mucin 1, a predominantly apical marker of the distal tubule,^23^^,^^24^ marks largely but not completely overlapping tubular segments (Figure 1e). Thus, immunodetection of the collagen IV α3α4α5 chains in the kidney exclusively mark the TBM of the distal tubule (in addition to the GBM and partially the Bowman capsule).

### Identification of Collagen IV–Producing Cells

**Figure 2:**
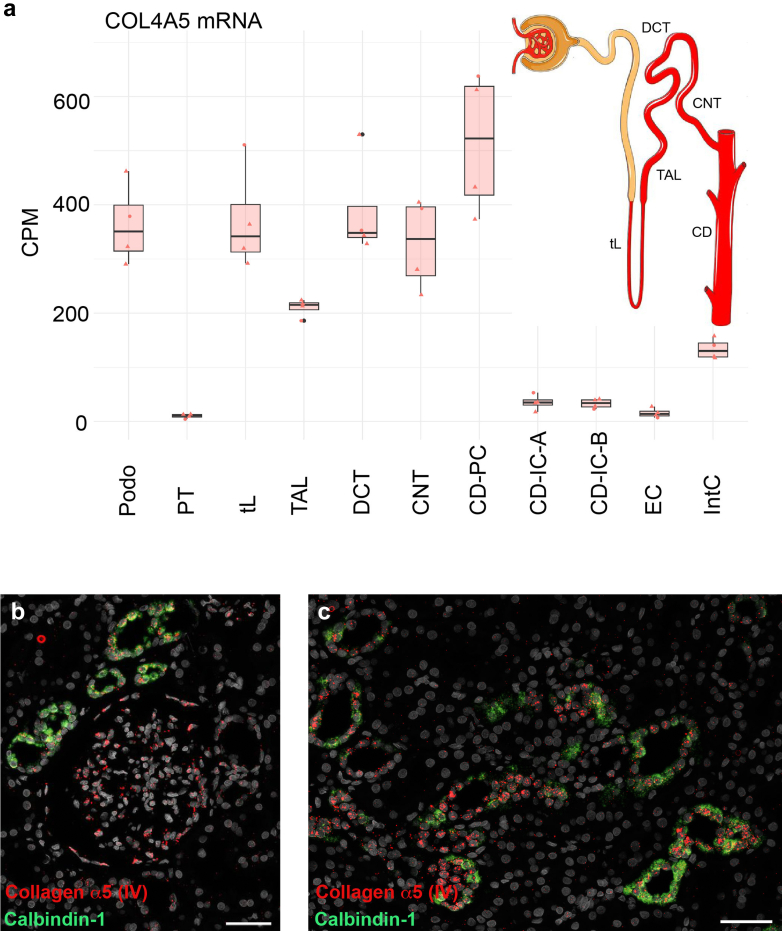
Collagen α5 (IV) mRNA expression by epithelial cells of the distal tubule. Single-nucleus RNA sequencing (a) data show strong COL4A5 mRNA expression (in CPM) in podo, tL, DCT, CNT, and CD-PC. The TAL and the IntC show a rather moderate expression. Near to no expression of collagen α5 (IV) can be seen in the PT, the intercalated cells type A and B of the collecting duct (CD-IC-A and –B) and ECs. Data was extracted from online material of Hinze *et al.*^21^ The scheme on the righthand side illustrates the positively expressing tubular segments for collagen α5 (IV) of the nephron in red (and named as abbreviated above). (b, c) RNA *in situ* hybridization of a healthy human kidney for collagen α5 (IV, red) and calbindin-1 (green), which marks the distal tubular segments of the DCT, CNT, and CD-PC (see also Supplementary Figure S2G). (b) Distinct glomerular cells are positive for collagen α5 (IV), which will mainly be podo in addition to parietal epithelial cells. (c) In the tubulointerstitium, most of the collagen α5 (IV) expression stems from the tubular epithelium, which mostly overlaps with calbindin-1. Scale bars correspond to 50 μm. CD-IC-A, intercalated cells type A of the collecting duct; CD-IC-B, intercalated cells type B of the collecting duct; CD-PC, principal cells of the collecting duct; CNT, connecting tubule; CPM, counts per million; podo, podocytes; DCT, distal convoluted tubule; EC, endothelial cells; IntC, interstitial cells; PT, proximal tubule; TAL, thick ascending limb of Henle; tL, thin limb of Henle.

We next aimed to determine exactly, along which section of the distal tubule the TBM shows deposition of the collagen IV (α345) molecule. First, we analyzed data from single-nucleus RNA-sequencing of the kidney from an established and accessible source.^21^ In Figure 2a, we show the single-nucleus mRNA expression of collagen α5 (IV) in the different cell types of the kidney, with podocytes being the best characterized source of synthesis for the GBM.^25^ From the thin loop of Henle to the collecting duct, collagen α5 (IV) shows mRNA expression in the same range to that of podocytes. Second, immunohistochemistry of collagen α5 (IV) with different tubular markers on consecutive sections confirmed the distribution of the protein in the respective TBM, starting from the thick ascending limb right through to the collecting duct (Supplementary Figure S1). Interestingly, in the collecting ducts, collagen α5 (IV) appears to be mainly expressed by principal cells, whereas collagen α3 and α4 (IV) appear to be mainly expressed by collecting ducts intercalated A and B cells (Supplementary Figure S2). Endothelial cells appear to express almost no transcripts of the collagen IV (α345) molecule and interstitial cells a low amount of all alpha chains.

The single-nucleus RNA-sequencing data already implicated substantial expression of the *COL4A3/4/5* genes by the tubular epithelial cells. This is affirmed when studying expression of collagen α5 (IV) by RNA *in situ* hybridization on human kidney tissue. Collagen α5 (IV)–expressing cells are dispersed throughout the glomeruli, most likely in podocytes and in parietal epithelial cells (Figure 2b). Importantly, tubular cells, most of which coexpress the distal marker calbindin-1 (Supplementary Figure S2G), clearly show collagen α5 (IV) expression (Figure 2c), as do very few interstitial cells (not shown).

**Figure 3:**
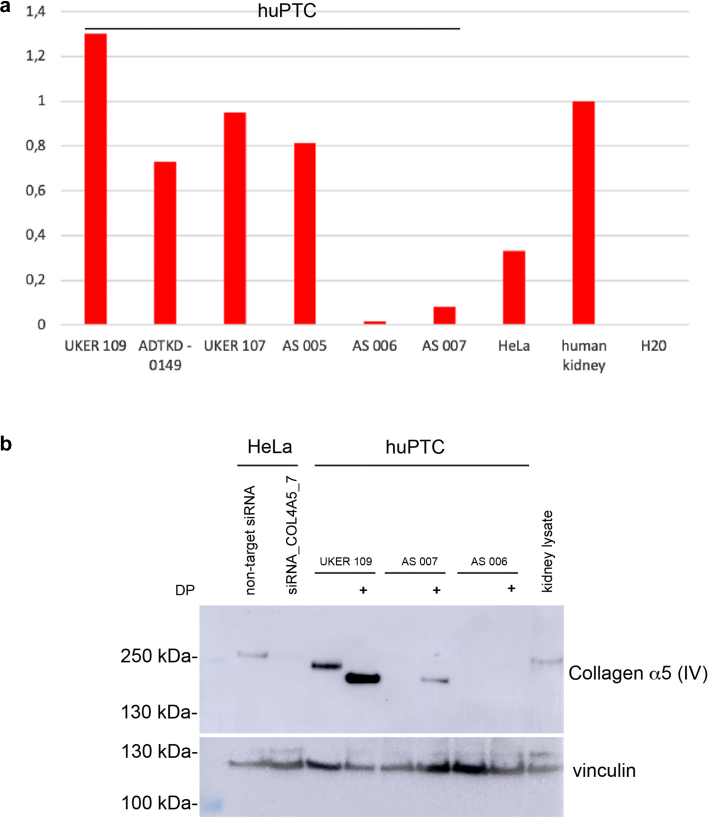
Renal tubular cells express collagen α5 (IV) in cell culture. Quantitative real-time polymerase chain reaction for mRNA expression of huPTC, immortalized HeLa cells, and human kidney lysate as a reference and arbitrarily set to a value of 1. The human kidney lysate and huPTC of a healthy human proband (UKER 109), a patient with autosomal dominant tubulointerstitial kidney disease-*MUC1* (ADTKD 0149) and a patient with renal tubular dysgenesis (UKER 107) show a strong RNA expression, as well as a male patient with the hypomorphic *COL4A5* variant c.1871G>A (AS 005). Interestingly, a female carrying a heterozygous duplication of 3 exons of *COL4A5* (Table 1) displays a marked reduction of mRNA expression (AS 007), whereas her hemizygous son with this variant shows almost no expression (AS 006). Immunoblotting for collagen α5 (IV) of whole cell extracts from HeLa cells and huPTC, (b) as well as a human kidney lysate, with vinculin as internal loading control. Positions of the molecular weight marker on the lefthand side. Collagen α5 (IV) in the kidney lysate and the untreated huPTC migrates just below the 250 kDa molecular weight marker. This species in the HeLa cell lysate migrates slightly slower, possibly indicating increased posttranslational modification. The signal in HeLa cells is completely abolished with knockdown by siRNA (COL4A5_7), similarly to the effect with other siRNA sequences against COL4A5 (Supplementary Figure S3A). In untreated huPTC lysates of a healthy proband (UKER 109) a strong species is visible for collagen α5 (IV), which is enhanced and migrates slightly faster upon treatment with the iron chelator DP, abolishing prolyl and lysyl hydroxylation and leading to partial intracellular accumulation (see Results section). Lysates of huPTC from a female carrying a heterozygous duplication of 3 exons of *COL4A5* (AS 007, Table 1) only show a detectable signal for collagen α5 (IV) upon treatment with DP, whereas her hemizygous son with this variant shows no expression under any condition (AS 006). DP, dipyridyl; huPTC, human primary tubular cells.

Next, we were interested to see whether renal tubular collagen IV expression is preserved in a tissue culture environment. huPTC from a healthy donor and patients with a hereditary kidney disease other than AS showed strong expression of COL4A5 mRNA measured by quantitative PCR (Figure 3a), as seen in human kidney tissue. Interestingly, the huPTC of a male patient with AS hemizygous for the hypomorphic *COL4A5* missense variant p.Gly624Asp (AS 005, Table 1)^26^ showed comparable mRNA expression to controls. In contrast, a mother heterozygous for a *COL4A5* duplication (AS 007; Table 1) and particularly her hemizygous son (AS 006) had strongly reduced mRNA expression, possibly because of RNA nonsense decay. Because very little data is available in the literature from immunoblotting of collagen α (IV) subunits, we aimed to detect collagen α5 (IV) in whole cell extracts. In HeLa cells, a species migrating at approximately 250 kDa is detectable, which can be effectively reduced by siRNA treatment (Figure 3b and Supplementary Figure 3A and B) or increased by iron chelation with dipyridyl (Figure 3b and Supplementary Figure 3C), which was associated with faster migration of the protein. After protein synthesis collagens are strongly modified, in part lysyl and prolyl hydroxylation by enzymes that are oxygen, iron, α-ketoglutarate, and ascorbate dependent.^27^ The prolyl hydroxylation in particular plays a role in stabilization of the triple-helical conformation of collagen.^28^^,^^29^ Iron chelation with dipyridyl has been shown to inhibit hydroxylation and thereby biosynthesis of the triple helix, leading to decreased cellular excretion and intracellular accumulation,^30^ which can be appreciated on the respective samples (Figure 3b).

### Ultrastructural Analysis of the TBM in AS

**Figure 4:**
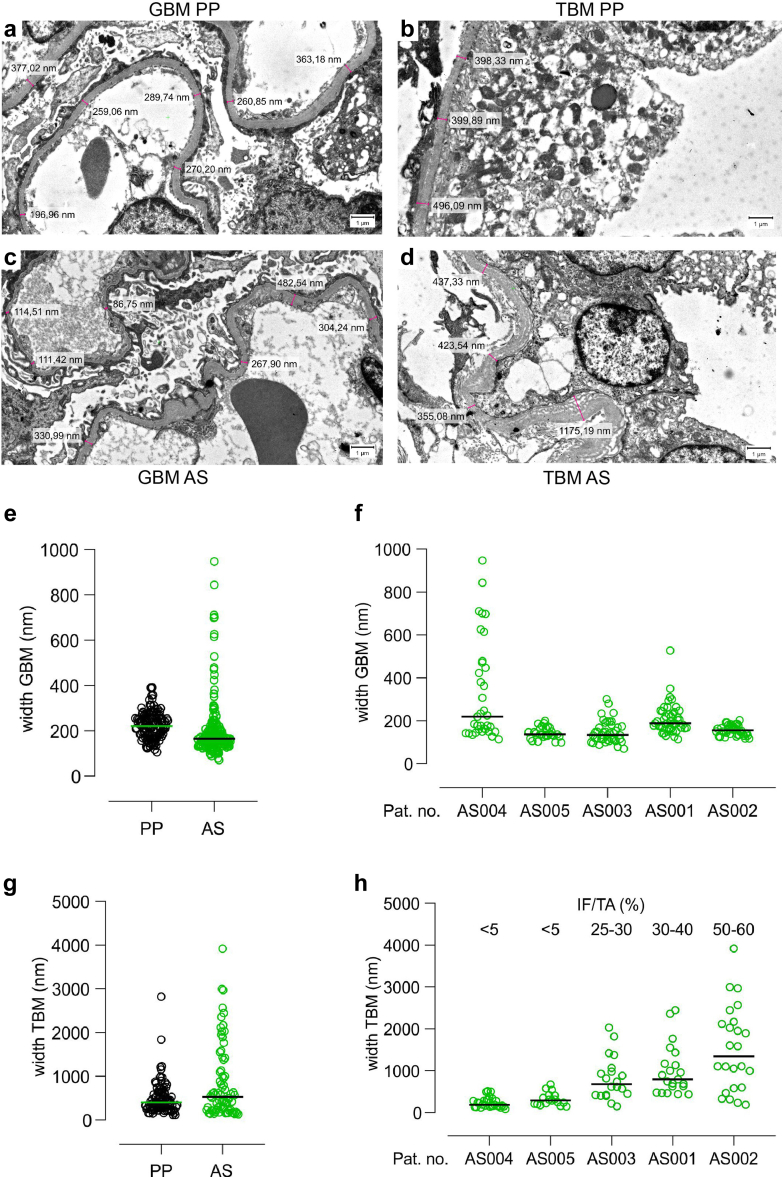
Ultrastructural basement membrane appearance in AS. Measurements of the width of GBM and distal TBM, respectively from clinical samples of kidney biopsies, processed for electron microscopy. Samples were from patients with AS or PP as control; *n* = 5 for both. (a–d) The pink bar and adjacent numbers represent the position and respective measurement in nm exemplifying the method of analysis. (c) Although the GBM in AS is rather thin (< 150 nm) in many positions, (d) the distal TBM is of variable width. (e) Quantification and comparison of all measurements grouped for PP and AS shows that the median for GBM is slightly thinner in AS than in PP (*P* = 0.4142, not significant), with somewhat more variability. (g) In contrast, the median width of the distal TBM is slightly thicker in AS than in PP (530.1 nm vs. 406.1 nm, respectively; *P* = 0.3484, not significant), again with more variability in AS. (h) Resolving all single measurements for AS individually clearly shows that the amount of IF/TA correlates with the width and variability of the TBM, (f) but interestingly not the GBM. AS, Alport syndrome; GBM, glomerular basement membrane; IF/TA, interstitial fibrosis and tubular atrophy; PP, primary podocytopathy; TBM, tubular basement membrane.

Pathologic variants of *COL4A3/4/5* in the germline lead to ultrastructural thinning or texture disruption of the GBM, which can be diagnostic in patient biopsies by electron microscopy.^5^ Therefore, we were interested in the putative changes of the TBM in AS and studied this in historical biopsies processed for electron microscopy. As control, we chose samples from patients with a classical glomerular disease, primary podocytopathy (PP, comprising minimal change glomerulonephropathy and focal segmental glomerulosclerosis). The glomerular and tubular BMs in PP showed little variability in width (representative images in Figure 4a and b). Although there is extensive literature on the width and appearance of the GBM - in AS (reviewed by Haas)^31^; to our knowledge there is no such data for the TBM. The width of the GBM and TBM in PP were in the (normal) median range of 220.4 and 406.1 nM, respectively (Figure 4e and g). In the cohort investigated here, the GBMs of patients with AS were mostly thinner (median 165.5 nM, representative image in Figure 3b), but showed a higher variability than in PP (*P* = 0.4142, not statistically significant, Figure 4c, e, and f). Of note, some of the distal TBM in AS showed irregularities of the BM (representative image in Figure 4d) with higher variability of TBM width as seen in patients with PP (Figure 4d, g, and h). Again, the mean TBM width (530.1 nM) was not statistically different from PP; however, measurements in AS samples were more widely scattered. Interestingly, when TBM width measurements are considered individually in relation to the degree of interstitial fibrosis and tubular atrophy it becomes clear that the irregularities in the distal TBM only appear when relevant interstitial fibrosis and tubular atrophy had developed in the kidney (Figure 4h). Thus, variability in TBM width seems to be closely correlated with the degree of tissue fibrosis, which needs to be tightly controlled for in further (and larger) studies. Of note, in the small cohort studied here, the width of the GBM did not appear to correlate with the grade of interstitial fibrosis and tubular atrophy (Figure 4f, h).

### Spatial Relationship of Distal Tubule and Fibrosis

**Figure 5:**
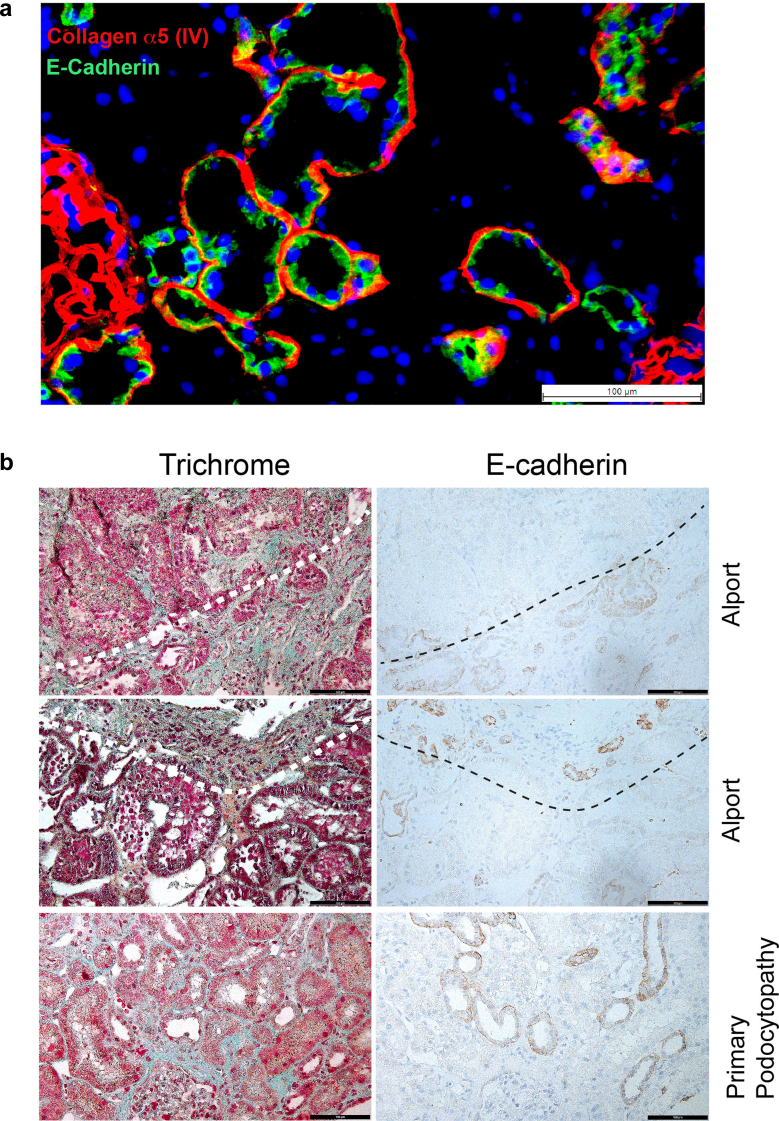
(a) Spatial relation of tubulointerstitial fibrosis to distal tubules in Alport syndrome. Immunofluorescence for collagen α5 (IV, red) and E-cadherin (green), a well-recognized marker of distal tubular segments of the kidney, shows that there is almost complete overlap of stained tubular segments in healthy kidney tissue sections. Because E-cadherin is predominantly membranous and collagen α5 (IV) extracellular in the TBM, there is some colocalization on a subcellular level at the contact sites (yellow). E-cadherin appears to be a suitable marker for the collagen IV α345–producing (distal) tubular segments, which mostly loose collagen IV α345 expression in AS. (b) Consecutive tissue sections from clinical kidney biopsies stained for Masson's Trichrome (left hand panels) or E-cadherin (right hand panels) from patients with Alport syndrome or primary podocytopathy. The Trichrome staining marks interstitial fibrosis in a blue-greenish color. Overall, the fibrotic areas in Alport syndrome can preferably be found around distal tubules, marked by E-cadherin staining (respective areas marked by the dotted lines). In the biopsies from primary podocytopathy, no clear spatial relationship to specific tubules could be recognized.

**Figure 6:**
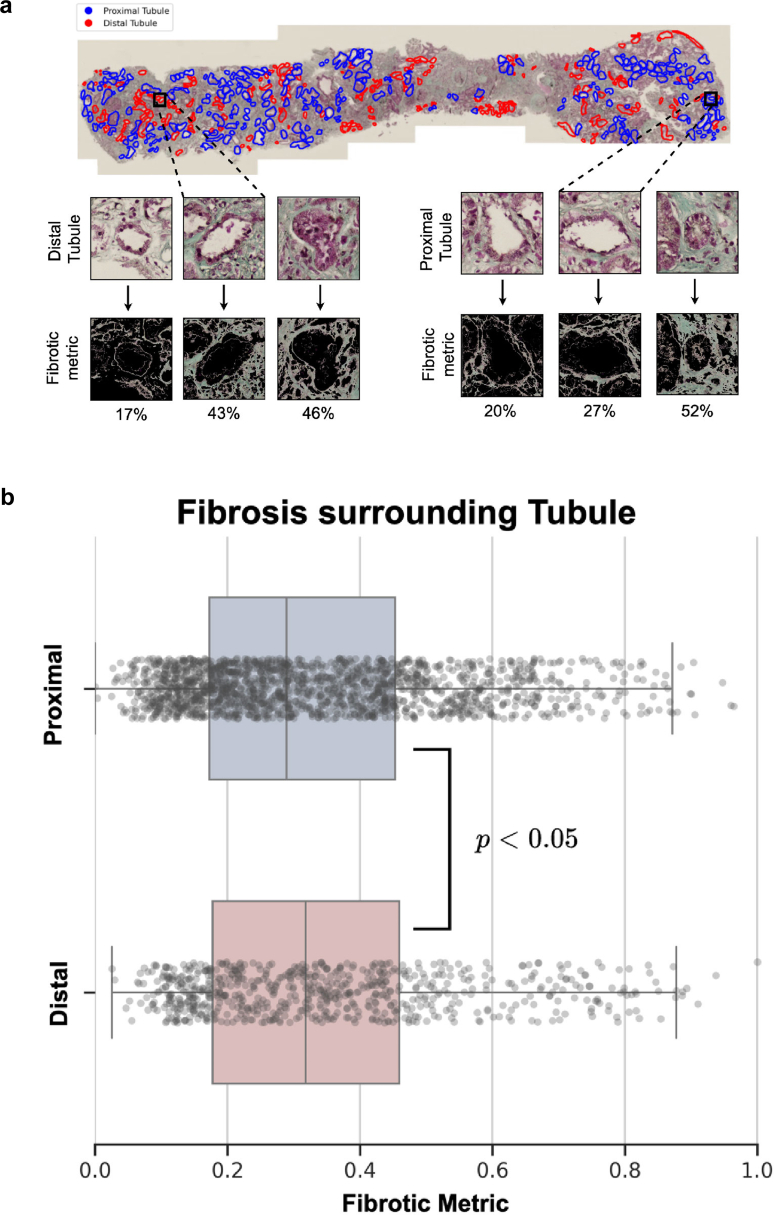
Computer-assisted quantification of peritubular fibrosis. (a) Whole-slide kidney biopsies from individuals with Alport syndrome were stained with Masson’s trichrome (upper panel) and E-cadherin (not shown). Tubules were annotated in Masson’s trichrome stained images as distal (red, for E-cadherin positive) or proximal (blue, for E-cadherin negative). For each tubule class, image tiles were sampled from the tubular center, fibrosis segmented using a standardized green-channel threshold, and a fibrosis metric was computed as percentage area per tile. (b) Distribution of the fibrosis metric computed for distal (*n* = 717) versus proximal tubules (*n* = 1724), box-and-whisker plots show median and interquartile range. The horizontal axis depicts the fibrotic metric, where 1.0 corresponds to 100%. We tested the *a priori* hypothesis that peritubular fibrosis is greater around distal than proximal tubules. Both an independent *t* test and Mann-Whitney U test supported this difference (*P* = 0.020 and *P* = 0.042, respectively).

Considering that genetically determined structural changes in the collagen composition of the TBM could be pathogenetically associated with tubulointerstitial fibrosis, we were next interested in whether there is a spatial relationship between areas of fibrosis and the distal tubules. Because the collagen IV (α345) molecule is absent or strongly diminished in kidney BM of patients with AS, we required a suitable marker for the respective tubular segments. Indeed, E-cadherin shows an almost complete overlap of the expression pattern with collagen α5 (IV) (Figure 5a and) Supplementary Figure 2H, which we therefore used for subsequent studies. Consecutive sections of biopsies from patients with Alport stained for E-cadherin and Massonʼs Trichrome indicate that interstitial fibrosis predominantly arises around distal tubules, where the TBM is structurally disturbed (Figure 5b, upper and middle panels). This spatial vicinity is not given in patient biopsies with a PP (Figure 5b, lower panel). However, these patients are mostly biopsied when there is very little fibrosis (Supplementary Table S2). To quantify the association between fibrosis and distal tubular segments objectively, we employed computer-assisted analysis on whole slide images of kidney biopsies from patients with AS (*n* = 4, Table 1). In Figure 6a, we depict a representative biopsy, with proximal (blue) and distal (red) tubules annotated (upper panels) and fibrosis quantified as the green-stained area within a tile surrounding each tubule in Masson’s Trichrome staining (lower panels). A total of 717 tiles surrounding distal tubules (defined as E-cadherin–positive) and 1724 surrounding proximal tubules (defined as E-cadherin–negative) were analyzed. The quantification results (Figure 6b) reveal that distal tubules are significantly encircled by more fibrosis than proximal tubules (*P* < 0.05), suggesting that tubulointerstitial fibrosis in AS originates primarily from the distal tubules. In contrast, matched controls with similar rates of interstitial fibrosis and tubular atrophy from patients with either hypertensive nephropathy or antineutrophil cytoplasmic autoantibody–associated vasculitis (Supplementary Table S3) did not show a difference of fibrosis rate between distal and proximal tubules (Table 2).

**Table 2:** Spatial fibrosis in different kidney diseases

Disease	Distal		Proximal		*P*-value	
	(*n* =)	Fibrotic metric	(*n* =)	Fibrotic metric	Independent *t* test	Mann-Whitney U test
Alport syndrome	717	32.8	1724	31.3	0.02	0.04
Hypertensive nephropathy	389	33.2	2328	36.6	0.99	0.99
ANCA-associated vasculitis	328	36.4	2142	35.7	0.14	0.86

ANCA, anti-neutrophil cytoplasmic autoantibody.

Automated fibrosis mapping by computer-assisted analysis was performed on whole kidney biopsy slides of patients with Alport syndrome and matched controls (hypertensive nephropathy and ANCA-associated vasculitis). The absolute number (*n* =) of analyzed tiles for distal (judged by E-cadherin positive staining) and proximal (judged by E-cadherin negative staining) are given for each investigated group (disease). The mean values are listed for each fibrotic metric, as well as the statistical results calculated by the independent *t* test and the Mann-Whitney U test, respectively.

## Discussion

More than 50 years ago, different investigators reported that fibrotic changes in the tubulointerstitial space are of utmost relevance for kidney function and prognosis of kidney failure,^32^^,^^33^ even in primary glomerular diseases.^34^ These observations were based on careful morphological studies in correlation with clinical data. However, the mechanisms of these effects remain elusive. Up to now, AS has been regarded as classical glomerular disease and the glomerular effects of the altered or lacking collagen IV (α345) molecule have been intensively studied (reviewed in Kruegel *et al.*).^5^ Although the effects of AS-causing variants on expression of collagen IV (α345) appear to be very similar in GBM and TBM (as described in the Introduction section), to our knowledge, there are no data on the consequences for the tubulointerstitial space. Interestingly, early studies demonstrated that preemptive ramipril in Col4α3 knockout mice had the ability to profoundly reduce tubulointerstitial fibrosis, which may be directly linked pathomechanistically.^35^ Our data presented herein imply that the tubulointerstitial space could directly contribute to the pathogenesis of AS and possibly accelerate the development of chronic kidney disease.

The milieu and function of the BMs in the glomerulus and the distal tubule of the kidney are different. In the former, high pulsatile shear stress is paired with high transmembranous filtration, where disease-causing collagen IV variants have been shown to lead to an increased susceptibility to endoproteolysis.^11^ The latter operates in a low-pressure environment with a high parallel flow of processed urine, but an energy-consuming solute exchange. Therefore, changes in the composition of the 2 BMs are likely to result in distinct molecular signals and pathways. However, as we have shown in a small number of patient biopsies using electron microscopy, structural changes of the TBM may result in a fibrotic signature via pathways that are yet to be identified. The molecular pathways leading to tissue fibrosis probably parallel those leading to kidney cysts, which appear to be a prominent and often bilateral finding in AS, which can be misdiagnosed as autosomal dominant polycystic kidney disease in some cases.^36^^,^^37^ These cysts originate from dedifferentiated tubular epithelial cells, which we have shown here, possibly produce most of the 3 components of the collagen IV α345 molecule deposited at the TBM. Thus, we speculate that the distal tubular epithelium is critical in the events for collagen IV synthesis in physiology, and in the pathophysiology of tubulointerstitial fibrosis in AS.

There is a considerable spectrum of collagen IV variants which potentially explain a certain phenotypic variability, from benign hematuria to slowly decreasing kidney function into adulthood and mild proteinuria, up to kidney failure with profound proteinuria in early adulthood, or even youth.^38^ A small number of patients may not show a completely typical phenotype and may be clinically misclassified into other disease spectra, such as focal segmental glomerulosclerosis (reviewed in Deltas *et al.*),^14^ hypertensive nephropathy, tubulointerstitial nephritis, or gout.^16^ Some cases may even phenocopy the hereditary, primarily tubulointerstitial disease autosomal dominant tubulointerstitial kidney disease.^15^^,^^16^^,^^39^ To date, it is unclear whether the phenotypic variability is solely dominated by the nature of distinct variants, or whether additional diseases or environmental influences push toward a certain phenotype. Interestingly, some variants appear to have discordant effects either on the glomerular or tubular compartment. For example, the *COL4A5* variant p.(Gly1030Ser) retains the expression in the GBM but not in the TBM^40^; a finding which has been reported in a mouse model with the glycine substitution p.(Gly1332Glu) as homozygous missense variant in Col4a3.^41^ Thus, certain pathogenic variants may retain (the glomerular) expression of the α345 (IV) molecule, which may cause a milder clinical course.^42^^,^^43^ The hypomorphic *COL4A4* variant p.(Gly545Ala) predisposes to a mild clinical course and a tubulointerstitial phenotype, resembling autosomal dominant tubulointerstitial kidney disease or hypertensive nephropathy.^39^ Therefore, in our opinion, the tubulointerstitial space contributes to the pathogenesis of AS, and may in a specific genetic context influence the phenotype slightly toward a tubulointerstitial and milder phenotype. This may result in atypical adult cases of AS, who are often misclassified and diagnosed with considerate latency.

An important limitation of our study is the small number of patients and tissues investigated. Indeed, the requirement of having a historic kidney biopsy and a conclusive genetic report, next to the personal contact to obtain the consent significantly reduced the number of eligible patients in our monocentric study. Furthermore, because of the clinical approach we were merely able to correlate data sets and not formerly prove the causal relation, which is particularly relevant for the association of tubulointerstitial fibrosis and the distal renal tubule. Thus, genetically modified animal models will be necessary, in which, according to our data, the distal tubular epithelium should be targeted.

In summary, our study adds a completely new aspect to the AS, a disease which has recently gained more attention because of the much-improved genetic recognition. In our opinion, the tubulointerstitium clearly deserves more attention in AS and should be investigated in larger cohorts and functional studies. Importantly, targeted drug development may need to consider drug delivery not only to the glomerulus, but also to the distal renal tubule.

## Disclosure

All the authors declared no competing interests.

## Supplementary Material

Supplementary File (PDF) **Supplementary Methods. Figure S1.** Segment-specific localization of collagen a5 (IV) in the kidney. **Figure S2**. Renal RNA expression of collagen IV genes and segmental markers of the tubular system. **Figure S3**. Establishment of collagen a5 (IV) immunoblotting. **Table S1**. Primary and secondary antibodies and their concentrations used in the different analyses. **Table S2**. Control probands with primary podocytopathy (PP). **Table S3**. Control probands for automated fibrosis mapping. STROBE Checklist.

## Footnotes

Supplementary File (PDF)

**Supplementary Methods**
**Figure S1**. Segment-specific localization of collagen α5 (IV) in the kidney. Immunohistochemistry on consecutive sections of healthy human kidney, which were stained for collagen α5 (IV, right hand panels), or tubular segmental markers, respectively (left hand panels). The proximal tubules were stained with an anti-Megalin antibody, the thick ascending limbs with an anti-Tamm Horsfall protein (THP) antibody, the distal tubules and collecting ducts with an anti-11ß-Hydroxysteroid-Dehydrogenase (11ß-HSD) antibody and the collecting ducts with an antibody against aquaporin 2 (AQP-2). Scale bars for size comparison of 100 μm are depicted in each right lower corner of the images. **Figure S2**. Renal RNA expression of collagen IV genes and segmental markers of the tubular system. Single-nucleus RNA sequencing data for collagen α3 (A), α4 (B), α1 (C), α2 (D) and α6 (E) (IV) chains in different tubular segments or cells: podocytes (Podo), proximal tubule (PT), thin limb of Henle (tL), thick ascending limb of Henle (TAL), distal convoluted tubule (DCT), connecting tubule (CNT), the principal cells of the collecting duct (CD-PC), the intercalated cells type A and B of the collecting duct (CD-IC-A and –B), the endothelial cells (EC), and the interstitial cells (IntC). Data are shown in counts per million and were extracted from online material of Hinze *et al.*^21^ **Figure S3**. Establishment of collagen α5 (IV) immunoblotting. Whole cell extracts from HeLa cells or human primary tubular cells (huPTC) of a healthy proband (UKER 109). (A) Knockdown with 4 different siRNA against COL4A5 showing a profound effect, respectively. (B) Treatment with the iron chelator dipyridyl increases protein amount and leads to faster migration of collagen α5 (IV). (C) Effect of dipyridyl and specific knockdown on cellular levels of collagen α5 (IV) in huPTC of a healthy donor. Vinculin was analyzed as internal control. **Table S1**. Primary and secondary antibodies and their concentrations used in the different analyses. **Table S2**. Control probands with primary podocytopathy (PP). **Table S3**. Control probands for automated fibrosis mapping STROBE Checklist.
