# Supplementary material for: Alport Syndrome is a Partial Tubulointerstitial Disease of the Kidney
Source: Kidney Int Rep. 2025 Nov 17;11(2):103694. doi: 10.1016/j.ekir.2025.11.019 (PMC12794056; doi:10.1016/j.ekir.2025.11.019)
Supplement: Supplementary File (PDF) — Supplementary Methods. Figure S1. Segment-specific localization of collagen a5 (IV) in the kidney. Figure S2. Renal RNA expression of collagen IV genes and segmental markers of the tubular system. Figure S3. Establishment of collagen a5 (IV) immunoblotting. Table S1. Primary and secondary antibodies and their concentrations used in the different analyses. Table S2. Control probands with primary podocytopathy (PP). Table S3. Control probands for automated fibrosis mapping. STROBE Checklist. [file mmc1.pdf]

SUPPLEMENTARY MATERIAL

Alport Syndrome is a partial tubulointerstitial disease of the kidney

Lisa Loderbauer[1], Karl X. Knaup[1], Daniel Reisenbüchler[2], Nicolas Kaiser[1, 3], Stephanie Naas[1], Karen Schneider[1], Florian J. Wopperer[1], Antje Wiesener[4], Francesca Pasutto[4], Mario Schiffer[1], Christoph Daniel[5], Katharina A. E. Broeker[6], Dorit Merhof[2], Maike Buettner-Herold[5] and Michael S. Wiesener[1]

Contents:

Supplementary Figure S1: Segment specific localization of collagen α5 (IV) in the kidney

Supplementary Figure S2: Renal RNA expression of collagen IV genes and segmental markers of the tubular system

Supplementary Figure S3: Establishment of collagen α5 (IV) immunoblotting

Supplementary Table S1: Primary and secondary antibodies and their concentrations used in the different analyses

Supplementary Table S2: Control probands with primary podocytopathy (PP)

Supplementary Table S3: Control probands for automated fibrosis mapping

Supplementary MethodsSTROBE Checklist

Supplementary information is available at KI Report's website.

Supplementary Figure S1

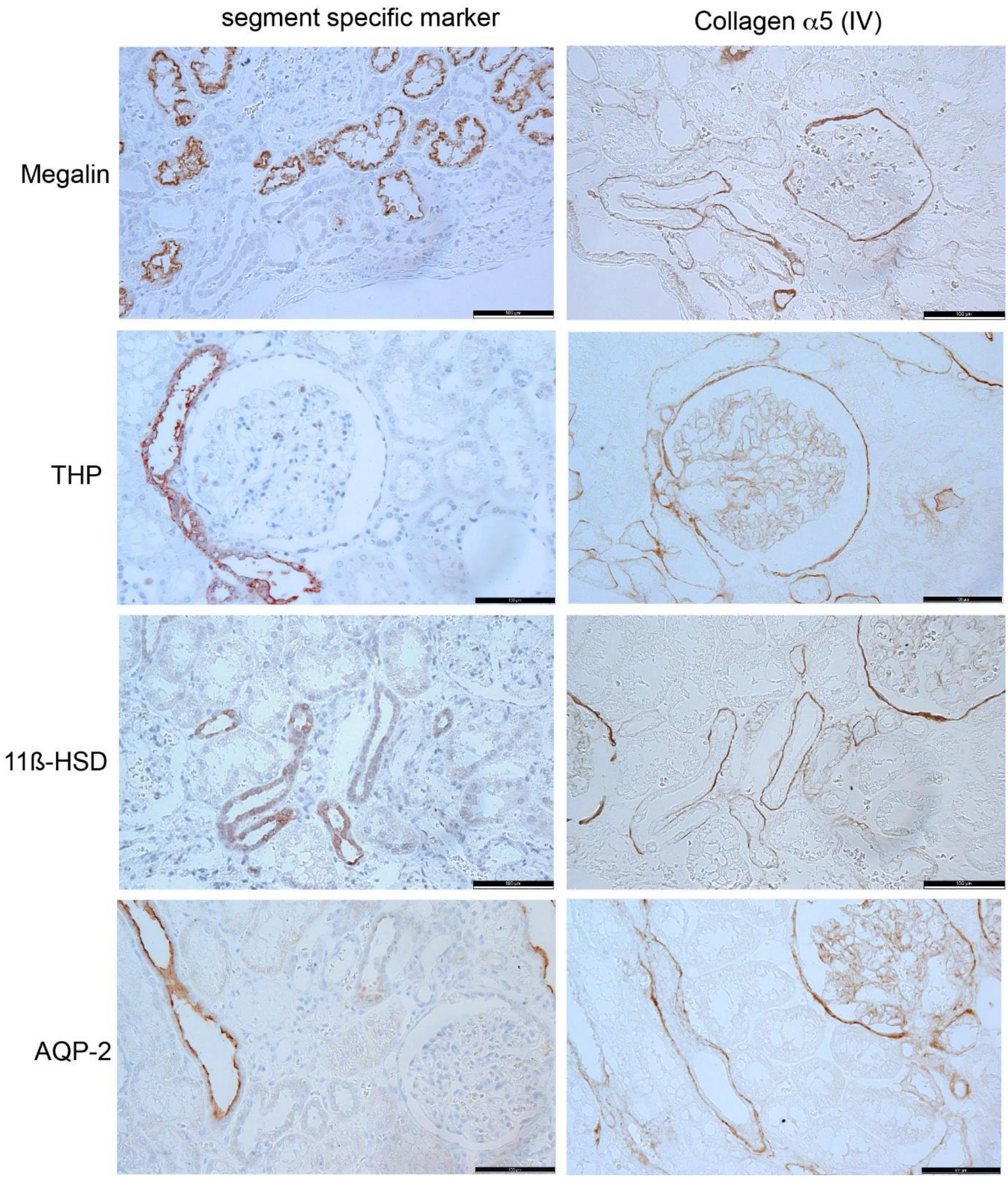

Supplementary Figure S1: Segment specific localization of collagen α5 (IV) in the kidney

Immunohistochemistry on consecutive sections of healthy human kidney, which were stained for collagen α5 (IV, right hand panels), or tubular segmental markers, respectively (left hand panels). The proximal tubules were stained with an anti-Megalin antibody, the thick ascending limbs with an anti-Tamm Horsfall protein (THP) antibody, the distal tubules and collecting ducts with an anti-11ß-Hydroxysteroid-Dehydrogenase (11ß-HSD) antibody and the collecting ducts with an antibody against aquaporin 2 (AQP-2). Scale bars for size comparison of 100 µm are depicted in each right lower corner of the images.

Supplementary Figure 2

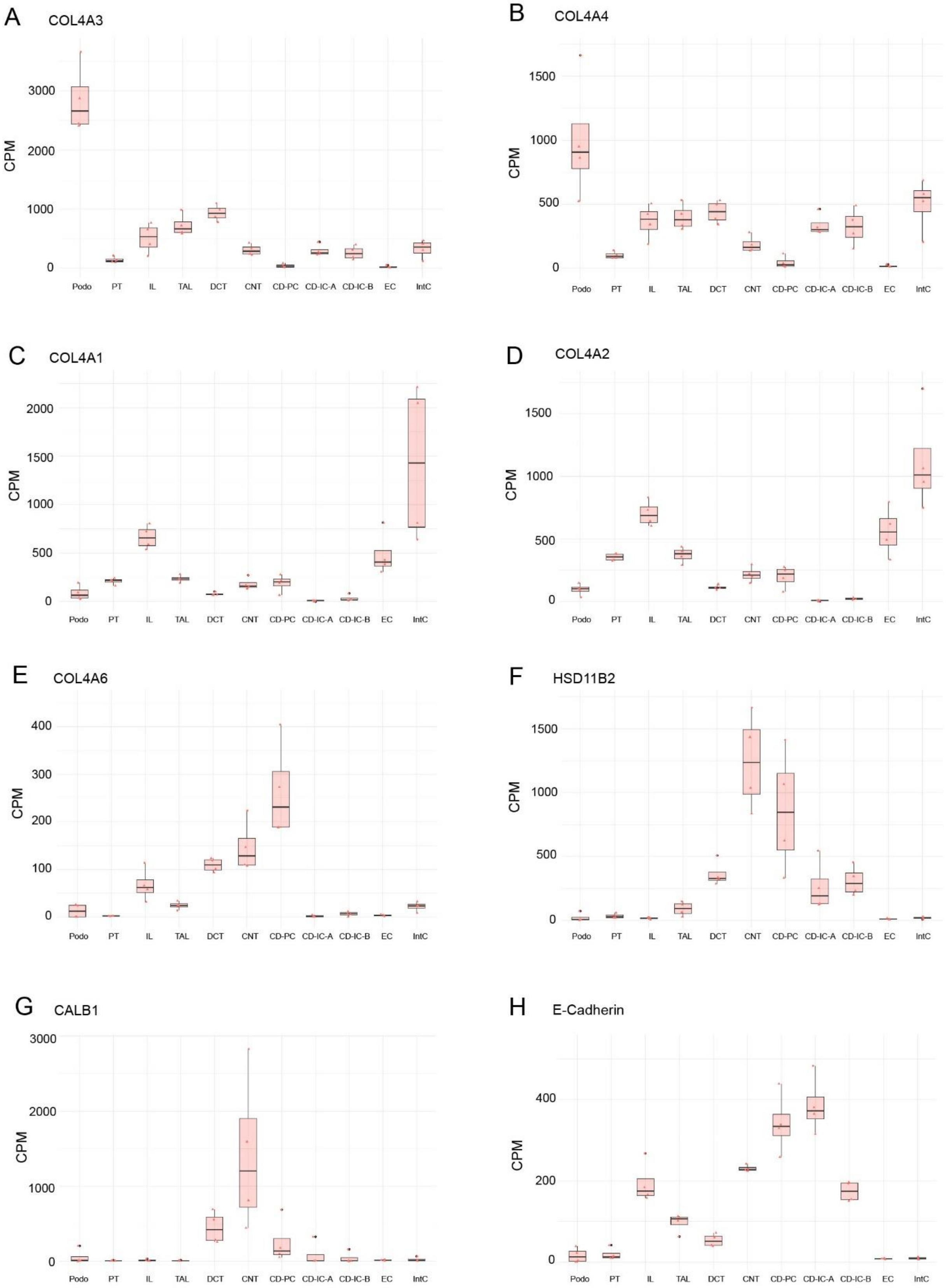

Supplementary Figure S2: Renal RNA expression of collagen IV genes and segmental markers of the tubular system

Single-nucleus RNA sequencing data for collagen α3 (A), α4 (B), α1 (C), α2 (D) and α6 (E) (IV) chains in different tubular segments or cells: podocytes (Podo), proximal tubule (PT), thin limb of Henle (tL), thick ascending limb of Henle (TAL), distal convoluted tubule (DCT), connecting tubule (CNT), the principal cells of the collecting duct (CD-PC), the intercalated cells type A and B of the collecting duct (CD-IC-A and –B), the endothelial cells (EC) and the interstitial cells (IntC). Data are shown in Counts per Million (CPM) and were extracted from online material of Hinze et al. [21].

Supplementary Figure 3

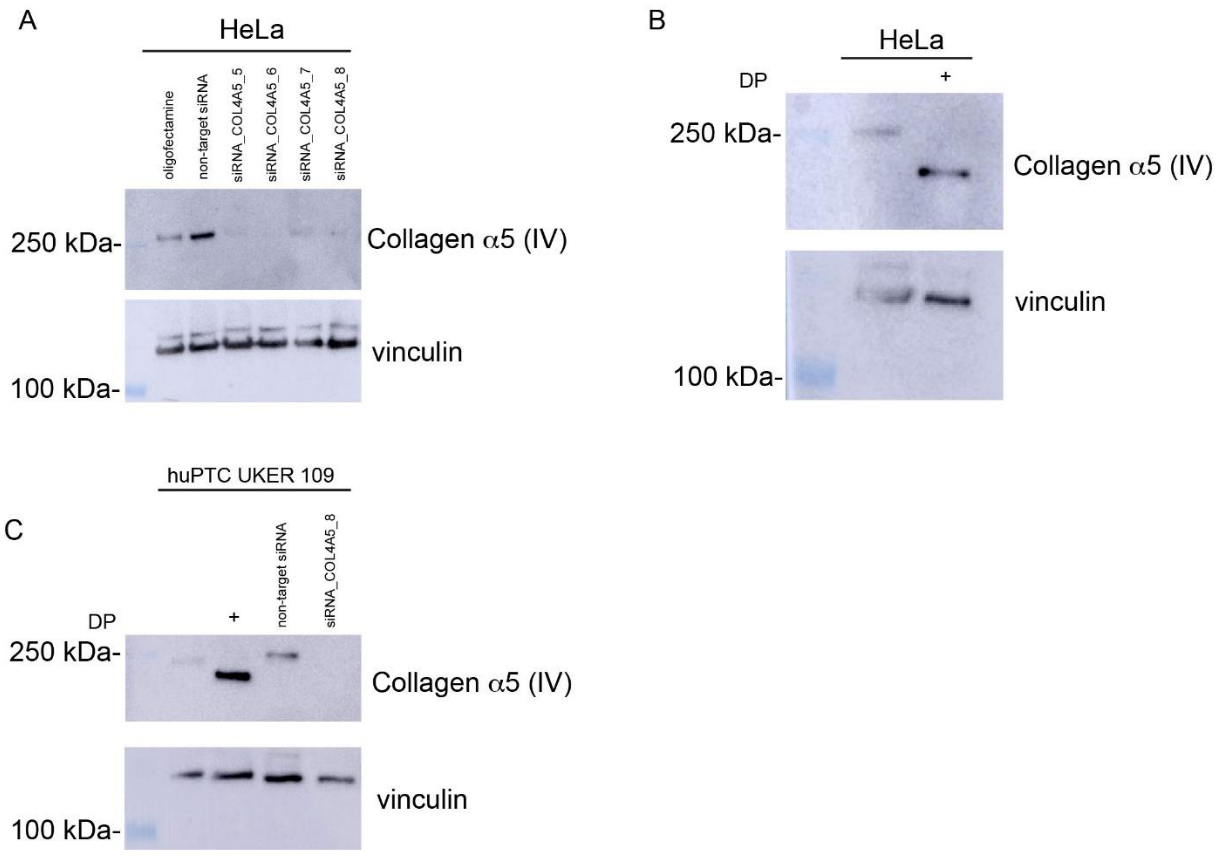

Supplementary Figure S3: Establishment of collagen α5 (IV) immunoblotting

Whole cell extracts from HeLa cells or human primary tubular cells (huPTC) of a healthy proband (UKER 109). A) Knockdown with four different siRNA against COL4A5 showing a profound effect, respectively. B) Treatment with the iron chelator dipyridyl increases protein amount and leads to faster migration of collagen α5 (IV). C) Effect of DP and specific knockdown on cellular levels of collagen α5 (IV) in huPTC of a healthy donor. Vinculin was analyzed as internal control.

Supplementary Table S1: Primary and secondary antibodies and their concentrations used in the different analyses

	**Company**	**species**	**concentration/dilution IB**	**concentration/dilution IHC/IF**
**primary antibody**				
Megalin	Acris (DM3613P), Herford, Germany	mouse	-	0,5µg/ml
11ß HSD	Millipore (AB1296), Darmstadt, Germany	sheep	-	100µl, 1 : 200
THP	MP Cappel (55140), Cambridge, UK	goat	-	6,95µg/ml
Aquaporin-2	Santa Cruz Biotechnology, Inc (sc-9880), Dallas, Texas	goat	-	0,4µg/ml
MUC1 WT (VNTR)	Cell Signaling (VU4H5), Danvers, USA	mouse	-	100µl, 1 : 100
COL4A3 H31	Chondrex Inc. (7076), Woodinville, U.S.A	rat	-	100µg/ml
COL4A4 H43	Chondrex Inc. (7073), Woodinville, U.S.A	rat	-	100µg/ml
COL4A5 H53	Chondrex Inc. (7078), Woodinville, U.S.A	rat	2µg/ml	10µg/ml
COL4A5 H52	Chondrex Inc. (7077), Woodinville, U.S.A	rat	-	10µg/ml
E-cadherin	BD Transduction Laboratories (610181)	mouse	-	0,8µg/ml
**secondary antibody**		**target/host**		
rb Anti-sheep IgG biotinylated	VectorLabs (BA-6000), Burlingame, USA	sheep/rabbit		3µg/ml
rb Anti-goat biotinylated	VectorLabs (BA-5000), Burlingame, USA	goat/rabbit		IHC 30µg/ml; IF 3µg/ml
rb Anti-rat biotinylated	VectorLabs (BA-4001), Burlingame, USA	rabbit/rat	-	10µg/ml
Anti-mouse biotinylated	Dako North America Inc. (K1500), Carpinteria, USA	-	-	ready-to-use reagent
Alexa Fluor 488	Invitrogen (A21042), Eugene, Oregon, USA	mouse/goat	-	4µg/ml
Alexa Fluor 594	Invitrogen (A11007), Eugene, Oregon, USA	rat/goat	-	4µg/ml
goat anti-rat HRP	Jackson ImmunoResearch Europe Ltd (112-036-008), Cambridgeshire, UK	rat/goat	0,4µg/ml	-

Supplementary Table S2: Control probands with primary podocytopathy (PP)

Proband´s pseudonym (ID), as well as their clinical data in respect to the stage of chronic kidney disease (CKD), age and grade of interstitial fibrosis and tubular atrophy (IF/TA)) at biopsy are listed.

PP	CKD	age at biopsy	IF/TA
PP 001	G1A3	25	<5%
PP 002	G1A3	19	<5%
PP 003	G1A3	34	<5%
PP 004	G3aA3	25	<5%
PP 005	G4A3	27	25-30%

Supplementary Table S3: Control probands for automated fibrosis mapping

Proband´s pseudonym (ID) with either hypertensive nephropathy (HNP) or ANCA-associated vasculitis (AAV), as well as their clinical data in respect to the sex, age and grade of interstitial fibrosis and tubular atrophy (IF/TA) at biopsy.

Disease	Proband-ID	Sex	Age at biopsy	IF/TA at biopsy (%)
HNP	HNP-001	m	43	50
HNP	HNP-002	m	46	15
HNP	HNP-003	m	54	50
AAV	AAV-001	m	72	20
AAV	AAV-002	m	65	20
AAV	AAV-003	f	36	20

Supplementary Methods

Cell Culture

HeLa cells were supplied from the German Collection of Microorganisms and Cell Cultures (DSMZ, Braunschweig, Germany).

For knockdown experiments the following siRNAs were used: siCOL4A5_5 (GeneGlobe ID SI03124212), siCOL4A5_6 (SI04141935), siCOL4A5_7 (SI04240243) and siCOL4A5_8 (SI04342016) (FlexiTube GeneSolution GS1287, Qiagen, Hilden, Germany).

Dipyridyl (DP) was added in a concentration of 100 µM for 18 hours on huPTC and HeLa cells.

Immunoblotting

For whole cell extracts, cells were lysed by sonication into extraction buffer (8 M urea, 10% glycerol, 1% SDS, 10 mM TrisHCl pH 6.8, protease inhibitor cOmplete™ (Roche, Mannheim, Germany)). Protein concentrations were measured with the DC Protein Assay (BioRad, California, USA) according to the manufacturer´s instructions. Protein separation was performed by SDS-PAGE and proteins were transferred to a PVDF membrane (Millipore, Bedford, MA, USA). 5% milk in TBS-T was used as blocking buffer for 1 h at room temperature after the transfer. For protein detection a primary antibody against collagen α5 (IV) was incubated at 4°C overnight, followed

by an HRP conjugated secondary antibody at room temperature for 30 min. Details of the antibodies are shown in Supplementary Table S1. Between incubations the membranes were washed 3x5 min with TBS-T and after incubation with the secondary antibody 4x10 min with TBS-T and 2 min with PBS. Signals were visualized using an ECL system (GE Healthcare, Munich, Germany).

In situ hybridization

5µm sections of formalin-fixed paraffin-embedded human kidney biopsies were analyzed using RNAscope Multiplex Fluorescent V2 Assay with RNAscope® probes Hs-COL4A5 (Cat. No. 461871) and Hs-CALB1-C2 (Cat. No. 422161-C2) as described in Wang et al. [19]. and Broeker et al. [20].

Polymerase chain reaction (PCR)

Cells were homogenized into TRK lysis buffer (peqGOLD Total RNA Kit, VWR life science, Darmstadt, Germany) for RNA extraction.

For reverse transcription cDNA synthesis was performed with High Capacity cDNA Reverse Transcription Kit from Thermo Fisher Scientific, according to the manufacturer´s instructions with 100 ng RNA.

The reverse transcription was performed in a thermal cycler using the following incubation conditions: 25°C for 10 min, 37°C for 2 h, 85°C for 5 min.

For quantitative PCR (qPCR) the Maxima SYBR Green/ROX qPCR Master Mix (Thermo Fisher Scientific) was used according to the manual with 2µl cDNA. After 5

min of centrifugation at 4°C and 1000 rpm the PRC was performed in StepOnePlus Real-Time PCR System (Applied Biosystems, Waltham, USA).

Incubation steps for PCR: denaturation at 95°C for 15 min. 30x: denaturation at 95°C for 1 min; annealing at 58°C for 1 min; extension at 72°C for 45 sec; final extension: at 72°C for 10 min.

Primers used (at 10nM) for COL4A5:

F: 5‘-CCA GGA ATA CCA GGT CCT AAA G-3‘

R: 5‘-GGA AGA CCT ACA TCA CCA TCT C-3‘

Immunohistochemistry

For antigen retrieval the formalin-fixed paraffin-embedded kidney biopsy sections were cooked in TRS (pH 6) over 2.5 min for E-cadherin and 5 min for Tamm Horsfall protein (THP), aquaporin 2 (AQP-2), Megalin and 11ß-Hydroxysteroid-Dehydrogenase (11ß-HSD) in a standard pressure cooker. The slides for collagen IV (α345) were cooked in 0.2N HCL (pH 0.9) using the digital decloaking chamber pro (Biocare Medical, Walnut Creek, California, USA) with a temperature of 115°C for 6 min.

The sections were blocked in 1% BSA in Tris-CSA (50mM Tris, 0.9% NaCl, 0.1% Tween-20, pH = 7.6) over 30 min for E-cadherin or in 3% H_2O_2 for 10 min for Megalin and AQP-2.

For THP and collagen IV (α5) the blocking step was performed using the CSA Kit (Dako North America Inc., Carpinteria, USA) with Avidin and Biotin for 20 min each, followed by a peroxidase (10 min) and protein (60 min) block. The peroxidase-

blocking solution from DAKO was used for 11ß-HSD over 10 min as well as 10% normal rabbit Serum in TBS-T as protein block for 30 min.

The primary antibodies (Supplementary Table S1) were added for 60 min at 37°C for 11ß-HSD, AQP-2, Megalin and over-night at 4°C for collagen α5 (IV), THP and E-cadherin.

The slides were incubated with the secondary antibodies (details in Supplementary Table S1) for 30 min at room temperature, before using the ABC amplification kit (Vectastain Elite ABC-HRP Kit, Vector Laboratories, Newark, USA) for 30 min.

The immunodetection was then performed with AEC+ (AEC Substrate Kit, Peroxidase (HRP), Vector Laboratories) in THP, AQP-2 and 11ß-HSD, DAB (DAB Substrate Kit, abcam, Cambridge, UK) for collagen α5 (IV) and Megalin or ImmPACT DAB (DAB Substrate Kit, Peroxidase (HRP), Vector Laboratories) for E-cadherin. For counterstaining (THP, AQP-2, 11ß-HSD and E-cadherin) hematoxylin was used for 1 min.

Between the incubation steps the slides were washed 3x5 min with PBS (collagen α5 (IV)), Tris-CSA (Megalin, AQP-2, E-cadherin) or TBS-T (THP, 11ß-HSD).

Co-localization studies

The cryosectioned tissues were fixed in 95% ethanol for 5 min at 4°C and washed 3x5 min in PBS, before antigen retrieval and permeabilization with 6M urea/0.1M Glycine HCl (pH 3.5) for 1 h at 4°C. For immunostaining the slides were incubated with primary antibodies for 1 h at 4°C and secondary antibodies for 1 h at room temperature. All details for antibodies can be found in Supplementary Table S1. DAPI

(Thermo Fisher Scientific, Waltham, USA) was added for nuclear DNA counterstaining in a concentration of 1:1000 for 2 min. For co-staining primary and secondary antibodies against E-cadherin and mucin 1 were added to the collagen α5 (IV) antibodies, respectively. The slides were incubated in a dark humidified chamber. PBS was used to wash the slides between the incubation steps.

(Re-)Analysis of single-nucleus RNA-sequencing (snRNA-seq) data

snRNA-seq data of control kidneys published by Hinze et al. [21]. were downloaded as cellranger-mapped count data and metadata including cell-type assignments from GEO (GSE210622). Control kidney data sets included snRNA-seq data from tumor-adjacent normal kidney tissues obtained from three patients undergoing tumor nephrectomy (Control-TN1, Control-TN2 and Control-TN3 corresponding to GSM6433700, GSM6433701 and GSM6433702) and three post mortem biopsies obtained from one individual without AKI (Control-15min, Control-60min, Control-120min corresponding to GSM6433703, GSM6433704 and GSM6433705). Since the control post mortem biopsies were acquired from the same individual, these data sets were merged. Data were processed as previously described using the R package Seurat (version 4.1.0) [22]. For each cell type and individual, a pseudobulk object was computed on raw counts and subsequently normalized to Counts per Million (CPM). Datapoints show the normalized pseudobulk expression of the indicated RNA transcript of interest per cell type and individual.

Analysis of Basement Membranes (BM) in Electron microscopy (EM)

For electron microscopy, kidney biopsies were fixed in 4% formalin buffered in 0.1M PBS pH 7.6, washed in 0.1 M PBS pH 7.6, treated with 0.5% OsO_4 for 60 min, and stained with 1% uranyl acetate in 70% ethanol. After the dehydration, tissue blocks were embedded in epoxy Araldite resin (Serva Electrophoresis GmbH, Heidelberg, Germany). 80 nm ultrathin sections were cut on a UC6 ultramicrotome (Leica, Wetzlar, Germany), rinsed in lead citrate buffer before analysis, and analyzed using a transmission electron microscope (Zeiss, Oberkochen, Germany) at 80kV. Glomerular and distal tubular BM of clinical biopsies were analyzed by EM from patients with primary podocytopathy (PP) or Alport Syndrome (AS); n = 5 for each. Tubular segments without brush border were classed as “distal” and the width of the BM measured in representative positions. Comparisons of the BM width measurements were conducted using a nested t-test and presented as a scatter dot plot. A finding was considered significant if the p-value would be smaller than 0.05.

STROBE Statement—checklist of items that should be included in reports of observational studies

	Item No	Recommendation	Page No
**Title and abstract**	1	(*a*) Characterization of the collagen IV alpha345 molecule in healthy human human kidney and tissues from patients with Alport syndrome	1-2
		(*b*) Provide in the abstract an informative and balanced summary of what was done and what was found	2
**Introduction**			
Background/rationale	2	Role of the collagen IV alpha 345 molecule in the tubular membrane completely unclear	4-5
Objectives	3	To define the expression and cellular source of the collagen IV alpha 345 molecule along the distal tubule, as well as its consequences in Alport syndrome. Hypothesis that alterations in basement membrane composition contribute to fibrosis and disease progression	4-5
**Methods**			
Study design	4	Immunodetection of collagen IV alpha 3, 4 and 5 chains and in situ hybridisation in human kidney biopsies: Tissue culture of primary cells. Immunoblotting and rt-PCR for COL4A5. Single-nucleus RNA sequencing and computer-assisted fibrosis mapping.	6-10 and Suppl. Material
Setting	5	Describe the setting, locations, and relevant dates, including periods of recruitment, exposure, follow-up, and data collection	Not applicable (NA)
Participants	6	(*a*) *Cohort study*— informed consent, existence of a historic kidney biopsy and genetic report<br>*Case-control study*—Give the eligibility criteria, and the sources and methods of case ascertainment and control selection. Give the rationale for the choice of cases and controls<br>*Cross-sectional study*—Give the eligibility criteria, and the sources and methods of selection of participants	6
		(*b*) *Cohort study*—For matched studies – (patients with primary podocytopathy)<br>*Case-control study*—For matched studies, give matching criteria and the number of controls per case	NA
Variables	7	Clearly define all outcomes, exposures, predictors, potential confounders, and effect modifiers. Give diagnostic criteria, if applicable	NA

Data sources/ measurement	8*	For each variable of interest, give sources of data and details of methods of assessment (measurement). Describe comparability of assessment methods if there is more than one group	NA
Bias	9	Describe any efforts to address potential sources of bias	NA
Study size	10	Explain how the study size was arrived at	NA
Quantitative variables	11	Explain how quantitative variables were handled in the analyses. If applicable, describe which groupings were chosen and why	NA
Statistical methods	12	(*a*) computer-assisted fibrosis mapping: independent t-test and Mann-Whitney U test<br>Comparison of basement membrane width: nested t-test	10<br>Suppl. Material, Methods
		(*b*) Describe any methods used to examine subgroups and interactions	NA
		(*c*) Explain how missing data were addressed	NA
		(*d*) *Cohort study*—If applicable, explain how loss to follow-up was addressed<br>*Case-control study*—If applicable, explain how matching of cases and controls was addressed<br>*Cross-sectional study*—If applicable, describe analytical methods taking account of sampling strategy	NA
		(*e*) Describe any sensitivity analyses	NA

Continued on next page

**Results**			
Participants	13*	(a) Report numbers of individuals at each stage of study—eg numbers potentially eligible, examined for eligibility, confirmed eligible, included in the study, completing follow-up, and analysed	NA
		(b) Give reasons for non-participation at each stage	NA
		(c) Consider use of a flow diagram	NA
Descriptive data	14*	(a) Patients affected with Alport syndrome with their genetic variant, age and CKD stage at time of biopsy	6, Table 1
		(b) Indicate number of participants with missing data for each variable of interest	NA
		(c) *Cohort study*—Summarise follow-up time (eg, average and total amount)	NA
Outcome data	15*	*Cohort study*—Report numbers of outcome events or summary measures over time	NA
		Case-control study—Report numbers in each exposure category, or summary measures of exposure	NA
		Cross-sectional study—Report numbers of outcome events or summary measures	NA
Main results	16	(*a*) Give unadjusted estimates and, if applicable, confounder-adjusted estimates and their precision (eg, 95% confidence interval). Make clear which confounders were adjusted for and why they were included	NA
		(*b*) Report category boundaries when continuous variables were categorized	NA
		(*c*) If relevant, consider translating estimates of relative risk into absolute risk for a meaningful time period	NA
Other analyses	17	Report other analyses done—eg analyses of subgroups and interactions, and sensitivity analyses	NA
**Discussion**			
Key results	18	tubulointerstitial space could directly contribute to the pathogenesis of AS and possibly accelerate the development of CKD	16
Limitations	19	Small sample and correlative data	18
Interpretation	20	Number of implications	16-18
Generalisability	21	Discuss the generalisability (external validity) of the study results	NA
**Other information**			
Funding	22	German Research Foundation (DFG, Projektnummer 509149993; TRR374, Project C4 to M.W. and Project C2 to M.B.-H. and C.D.) as well as personal grants to the Else Kröner-Fresenius-Stiftung and the Eva Luise und Horst Köhler Stiftung – Project No: 2019_KollegSE.04: “Research Center On Rare Kidney Diseases” (RECORD) to N.K. and	25

	the Interdisciplinary Center for Clinical Research (IZKF) of the University Hospital Erlangen to L.L.

*Give information separately for cases and controls in case-control studies and, if applicable, for exposed and unexposed groups in cohort and cross-sectional studies.

Note: An Explanation and Elaboration article discusses each checklist item and gives methodological background and published examples of transparent reporting. The STROBE checklist is best used in conjunction with this article (freely available on the Web sites of PLoS Medicine at http://www.plosmedicine.org/, Annals of Internal Medicine at http://www.annals.org/, and Epidemiology at http://www.epidem.com/). Information on the STROBE Initiative is available at www.strobe-statement.org.
